# Magnetic resonance findings in sellar and suprasellar tuberculoma with hemorrhage

**DOI:** 10.4103/2152-7806.72624

**Published:** 2010-11-20

**Authors:** Puneet Mittal, Sarika Dua, Kavita Saggar, Kamini Gupta

**Affiliations:** Department of Radiodiagnosis, Dayanand Medical College and Hospital, Ludhiana, Punjab, India

**Keywords:** Hemorrhage, MRI, sellar, tuberculoma

## Abstract

**Background:**

Tuberculosis is endemic in many counteries like India. It can infect any site in the central nervous system. However, islolated involvement of the sellar and suprasellar region is rare. Sellar tuberculoma with hemorrhage is even more rare. We present magnetic resonance (MR) findings in case of sellar and suprasellar tuberculoma with hemorrhage.

**Case Description:**

A 40-year-old female patient presented with a 1-month history of persistent headache and blurred vision on the left side. A contrast-enhanced MR study revealed peripherally enhancing sellar and suprasellar mass with hemorrhage with compression of the left half of the optic chiasma. There was also evidence of infundibular thickening and enhancement of the adjacent dura. The mass was approached through a transphenoidal approach and was partially resected. Subsequent histopathology was suggestive of tuberculosis. The patient was put on anti-tubercular therapy. Patient reported significant improvement in symptoms. Follow-up MR done 8 months later confirmed complete regression of the mass.

**Conclusion:**

Because of its rarity, sellar tuberculoma is seldom considered in the differential diagnosis and is often mistaken for pituitary macroadenoma, which is the most common tumor in this region. Although rare, presence of infundibular thickening and enhancement of the adjacent dura should suggest the presence of a granulomatous lesion like tuberculoma.

## INTRODUCTION

Intracranial tuberculosis is a common infection is India. However, involvement of the pituitary gland is very unusual. There are only isolated reports. To our knowledge, there are about 51 case reports of sellar tuberculosis.[1,6,7,10,11,13–15] Some of these had associated suprasellar or parasellar involvement while some were purely sellar. Most of these cases have been reported from India. To our knowledge, there are only two reports of pituitary tuberculosis with hemorrhage.[2,5] Thickening and enhancement of the pituitary stalk has been reported to be a useful imaging finding, although it can be seen in other conditions like sarcoidosis, lymphocytic hypophysitis, lymphoma and eosinophilic granuloma. Histopathology is the usual mode of making the diagnosis.

## CASE REPORT

A 40-year-old female patient presented with persistent headache and blurring vision on the left side for 1 month. She had no history of trauma or menstrual complaints. She was on hormonal replacement therapy for thyroid hormone deficiency for 2 months. She had no history or evidence of tuberculosis elsewhere in the body. A contrast-enhanced magnetic resonance (MR) study was performed. A sellar mass with suprasellar extension was seen. It appeared isointense, with multiple hyperintense areas on T1W images. Suprasellar extension of the mass was bulging to the left of the midline and was causing compression of the left side of the optic chiasma [Figure 1a]. The mass appeared heterogeneously hyperintense on T2W images, with multiple hypointense areas [Figure 1b] that bloomed on the gradient echo (GRE) images [Figure 1c]. On the post-contrast images, it showed peripheral rim-like enhancement. The pituitary stalk was thickened and enhancing. Adjacent dura also showed enhancement. [Figure 2a and b]. Pre-operative diagnosis was pituitary macroadenoma with hemorrhage. Subsequently, the patient underwent transphenoidal surgery with partial piece meal resection of the mass. Histopathology [Figure 3] confirmed the presence of hemorrhage and necrosis. Many epitheloid cells were seen in the periphery with lymphocytes and plasma cells, indicating granulomatous lesions. No malignant cells or adenoma cells were seen. Histology was suggestive of tuberculosis of the pituitary. The patient was put on anti-tubercular treatment. The patient returned for a follow-up scan after 8 months. On follow-up MR, the mass had completely regressed thus confirming the diagnosis; however, there was persistent mild thickening of the pituitary stalk [Figure 4a and b].

**Figure 1a:**
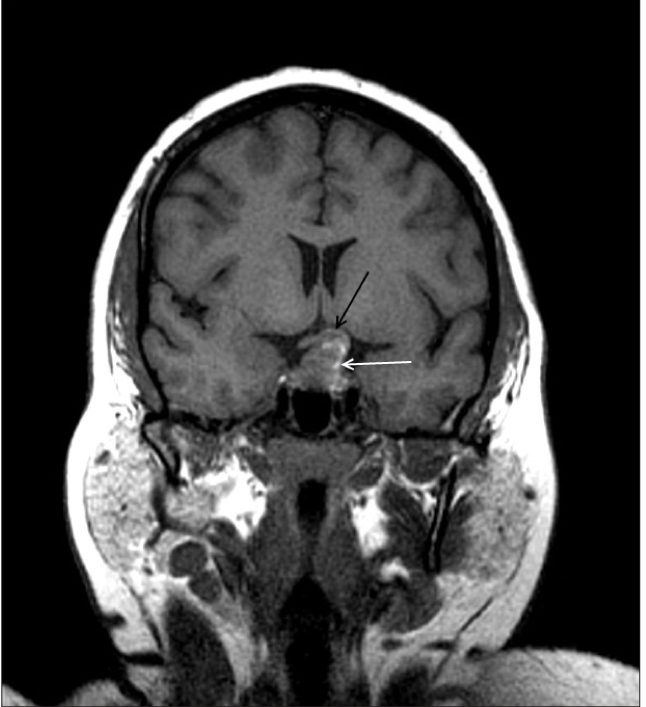
Coronal T1W MR image shows a predominantly isointense mass with few hyperintense areas (white arrow). It is bulging to left of midline and causing compression of left half of optic chiasma (black arrow)

**Figure 1b:**
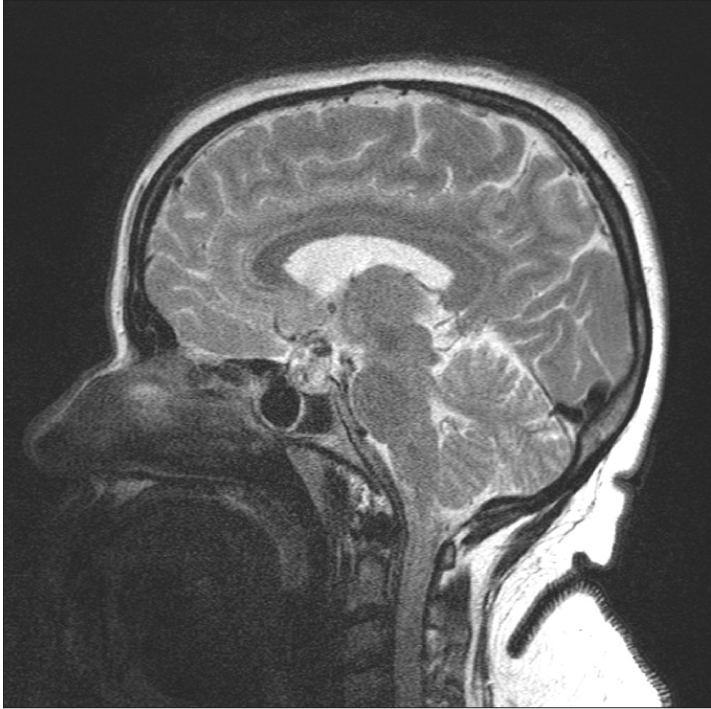
Sagittal T2W MR (Figure 1b) image shows a heterogeneous mass with multiple hypointense areas which bloom on GRE image (Figure 1c, white arrow) indicating haemorrhage.

**Figure 1c:**
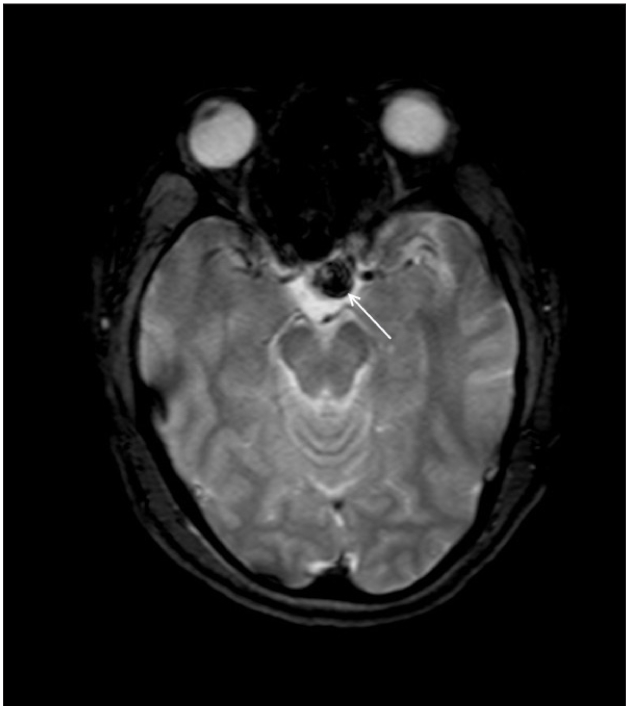
Sagittal T2W MR (Figure 1b) image shows a heterogeneous mass with multiple hypointense areas which bloom on GRE image (Figure 1c, white arrow) indicating hemorrhage.

**Figure 2a and b:**
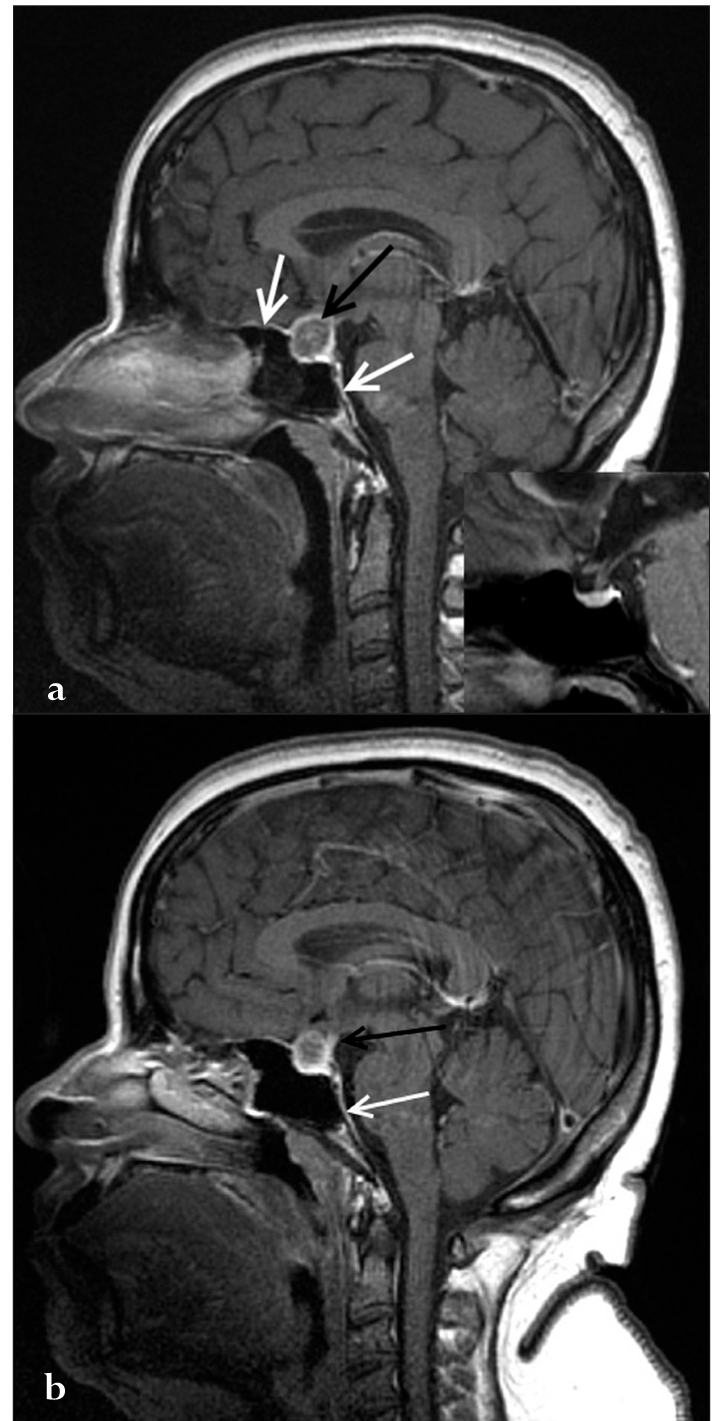
Sagittal post-contrast image showing rim enhancement (black arrow in a) in the mass. The adjacent dura also shows enhancement (white arrows in a and b). The inset shows a normal pituitary on the post-contrast images for comparison. The sagittal post-contrast image (b) shows a thickened and enhancing pituitary stalk (black arrow in b).

**Figure 3a:**
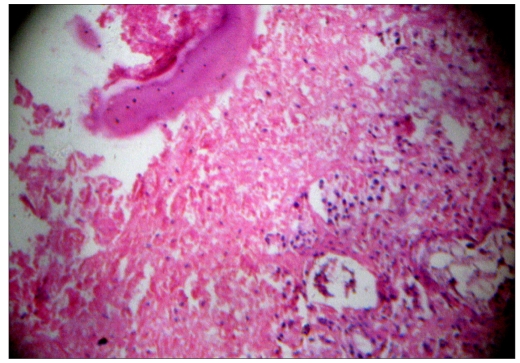
Photomicrograph (3a) shows areas of necrosis. Many epitheloid cells were seen in the periphery with lymphocytes and plasma cells indicating granulomatous lesions. Normal pituitary tissue is seen elsewhere (3b). No adenoma component is seen. (H & E, ×50)

**Figure 3b:**
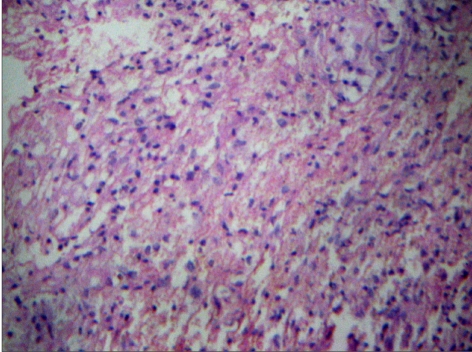
Photomicrograph (3a) shows areas of necrosis. Many epitheloid cells were seen in the periphery with lymphocytes and plasma cells indicating granulomatous lesions. Normal pituitary tissue is seen elsewhere (3b). No adenoma component is seen. (H & E, ×50)

**Figure 4a:**
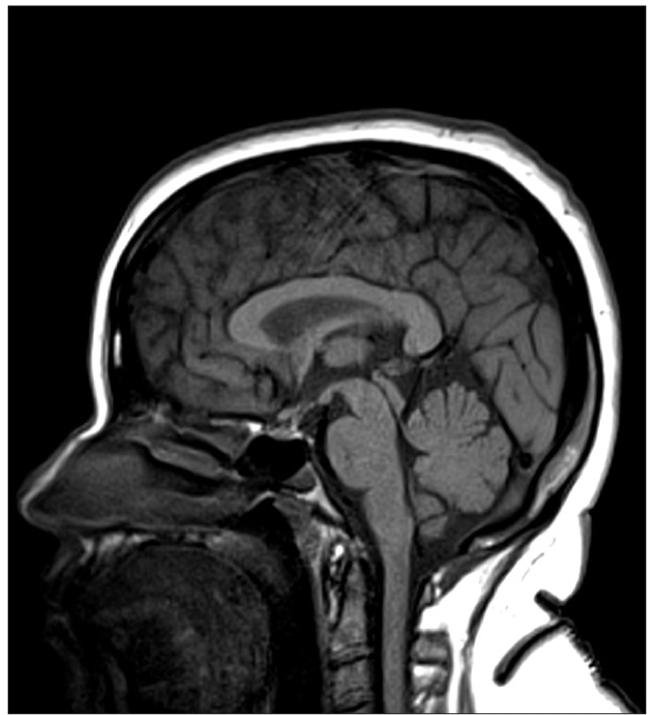
Sagittal (Figure 4a) and coronal T1W (Figure 4b) images at follow up show that the mass has largely disappeared. However there is persistent mild thickening of pituitary stalk

**Figure 4b:**
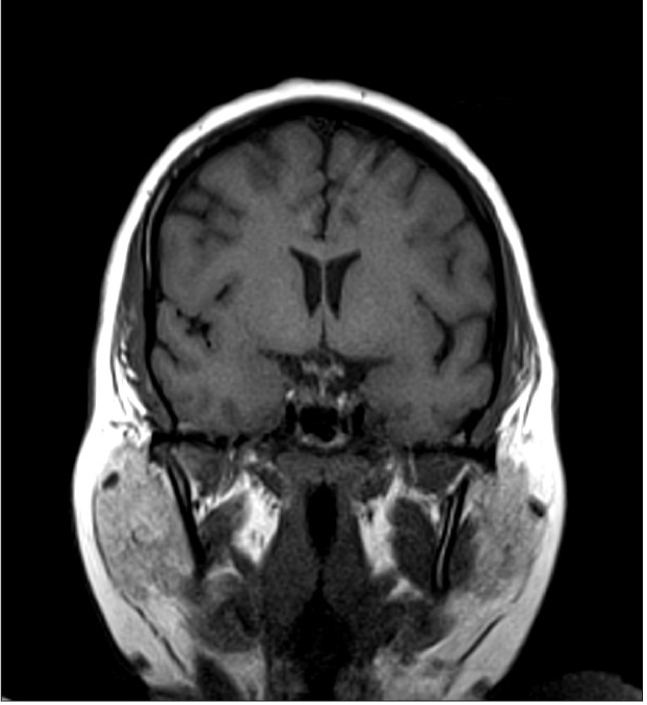
Sagittal (Figure 4a) and coronal T1W (Figure 4b) images at follow up show that the mass has largely disappeared. However there is persistent mild thickening of pituitary stalk.

## DISCUSSION

Sellar tuberculosis is a very rare infection. It was first described long back in 1940 by Coleman *et al*.[4] Since then, there have been isolated case reports. The largest series of 18 cases has been reported by Sharma *et al*.[12] There is a relative predominance of female cases.[7] The most common presenting features are headache, visual disturbances and hormonal disturbances.[2]

Pituitary apoplexy is a condition characterized by acute neurological symptoms due to hemorrhage in the pituitary. Pituitary adenoma is the predominant cause in a large proportion of the cases, and most of these are non-functioning macroadenomas.[8] Other reported causes include exogenous estrogen administration, pregnancy, surgery, myocardial infarction, severe infections and administration of anticoagulant drugs. The majority of the patients present without any apparent cause, which could be due to ignorance of the precipitating causes.[3]

On MR imaging, tuberculomas closely mimic pituitary adenoma. They present as a largely isointense mass on T1 and T2W images with heterogeneous areas. Uniform, heterogeneous as well as peripheral rim-like enhancement is described, the non-enhancing areas being attributed to caseation necrosis. Other findings that have been considered useful include thickening of the pituitary stalk and enhancement of the adjacent dura.[7,14]

Our index case showed a heterogeneous mass on T1W images with multiple hyperintense areas which bloomed on the GRE images, indicating hemorrhage. It was heterogeneous, predominantly hypointense on T2W images. On post-contrast images, it showed ring-like enhancement. The pituitary stalk was mildly thickened. The adjoining dura also showed enhancement. Although findings indicate the possibility of some granulomatous lesion, diagnosis of tuberculosis was not considered because of its rarity and due to the presence of the associated hemorrhage. A follow-up scan after surgery and antitubercular therapy showed that the mass had largely regressed. However, thickening of the pituitary stalk was still persistent, which has also been reported previously.[2]

It has been suggested that in any sellar mass with thickening and enhancement of the pituitary stalk, diagnosis of tuberculosis should be considered especially in places where tuberculosis is endemic. Thickening of the pituitary stalk can also be seen in other conditions like sarcoidosis, lymphocytic hypophysitis, lymphoma and eosinophilic granuloma.[9,14]

Hemorrhage in a sellar tuberculoma is extremely rare, with only two case reports in the literature.[2,5] The first case was reported by Arunkumar and Rajsekhar[2] in 2001. Hemorrhage is supposed to be secondary to vasculitis. Both these patients presented with acute symptoms thus enabling the diagnosis of pituitary apoplexy. On the other hand, symptoms in our patient were more subacute, which could be secondary to repeated small hemorrhages.

In conclusion, sellar tuberculoma is a rare condition and is difficult to diagnose on imaging. The presence of hemorrhage is even more atypical. Findings that can be useful in diagnosis include ring enhancement, thickening of the pituitary stalk and enhancement of the adjacent dura. These features can help one to consider the possibility of some granulomatous condition and guide further investigations to reach the correct diagnosis.
